# Carbon sphere-zinc sulphate nanohybrids for smart delivery of zinc in rice (*Oryza sativa* L)

**DOI:** 10.1038/s41598-021-89092-9

**Published:** 2021-05-04

**Authors:** Muthuraman Yuvaraj, Kizhaeral Sevathapandian Subramanian

**Affiliations:** 1 Department of Nanoscience and Technology, Tamil Nadu Agricultural University, Coimbatore, Tamil Nadu India; 2 Tamil Nadu Agricultural University, Coimbatore, Tamil Nadu India

**Keywords:** Plant sciences, Nanoscience and technology

## Abstract

The laboratory research was attempted to find nano zinc fertilizer utilizing the carbon sphere as a substrate. Typically the encapsulation techniques are followed in the drug delivery system, the limited work was done in nano-based zinc micronutrient for slow release of nutrients to crop. The use efficiency of zinc micronutrients in the soil is only less than 6 percentage. In universal, the deficiency of zinc micronutrients causes malnutrition problems in human beings, especially in children. After considering this problem we planned to prepare zinc nano fertilizer by using the carbon sphere for need-based slow release and increase the use efficiency of zinc micronutrient in soil. In this study we synthesis the carbon sphere nanoparticle after the formation of carbon sphere the zinc sulphate was loaded and characterized by utilizing Scanning Electron Microscopy, Surface Area and Porosity, X-ray diffraction analysis, Fourier Transform Infrared Spectroscopy, Transmission Electron Microscopy. The research result shows that the nano carbon sphere was excellently loaded with zinc sulphate to the tune of 8 percentage and it was confirmed by Energy dispersive X-beam spectroscopy. The zinc loaded carbon sphere release nutrient for a prolonged period of up to 600 h in the case of conventional zinc sulphate zinc release halted after 216 h in percolation reactor studies. The zinc nano fertilizer is recommended in agriculture to increase zinc use efficiency, crop yield without any environmental risk.

## Introduction

In worldwide fifty percentage of arable land shows a decrease in crop yield because of zinc inadequacy^1,2^. Zinc is a trace essential mineral to all forms of life science of its vital role in gene expression, cell expansion, and replication it is a catalytic and structural protein cofactor in various enzymes in plants^3,4^. About, 49% of arable land in worldwide is affected by zinc deficiency. The calcareous, alkaline soil, intensively cropped soils, sandy soil and high phosphorous soils having high Zn deficiency^5,6^. The application of zinc fertilizer in the soil the plant consumes only 2–5 percent remaining will get fixed into the soil and unavailable to crop^7,8^. In India, most of the region soils are highly deficient to zinc due to over or imbalanced application of fertilizer and unused organic manure which cause decreased crop yield^9^. Nowadays the nanotechnology plays a vital role in slow-release fertilizers in agriculture. Nanotechnology the potential to bring the next revolutionary breakthrough in agriculture-biased natural resource management^10^. It has ushered as a new interdisciplinary venture-field by meeting science and engineering into agriculture and food systems^11^.

The main carrier of our research is carbon spheres and it plays a vital role in energy storing properties, high surface area and biocompatibility^12^^.^ High chemical inertness, high specific surface area, thermal insulation, low active density and high compressive strength^13^. drug release, active substance encapsulation. The carbon sphere is used as electrodes^14^, supports for catalyst^15^ capsules for magnetic nanoparticles^16^, templates for making other hollow materials^17^, gas storage media, etc. Different method is used to synthesis carbon like pyrolysis method^18^, medial-reduction route^19^ solvothermal method^20^, and chemical vapor deposition method^21^. Among this method, we follow a carbon sphere synthesis from glucose^12^. Despite these materials, only limited research is undertaken in agriculture and we have discussed here fewer studies related to the carbon sphere in agriculture.

Advancement of smart delivery systems a novel method for the target release of fertilizer has many benefits. The encapsulated fertilizer shows better stability and increases nutrient use efficiency^22^. Control release fertilizers are encapsulated by using graphene oxide films with potassium nitrate, fortification by graphene oxide suggestively covers the controlled release of fertilizer in low-cost production^6,23,24^.

The use of carbon nano molecule 0–125 mg/pot on (*Nicotiana tabacum* L) plants brought about improved development at various stages as contrasted and the plant development acquired by utilizing ordinary fertilizer. These researchers additionally revealed that the utilization of carbon nano molecule expanded the substance of nitrogen and potassium in (*Nicotiana tabacum)* plant^25^. The wheat (*Triticum aestivum* L) crop was 50 mg L^−1^ treated with carbon nanoparticle in the soil for 20 days it will help shoot and root lengths improved up to three folds compared with the controls^26,27^.

In India at Tamil Nadu Agricultural University, Coimbatore, a few nano-zeolite related studies undergone with Nitrogen^28,29^ Phosphorous, and Potassium^30^ Sulphur^31,32^ Zinc^33^ have been combined and tried in a different form. Based on this literary works there is no research done with zinc micronutrient so we carried out and developed carbon sphere-based nano zinc fertilizer to improve the productivity and keep up ecofriendly. These studies suggest that Zn fertilization is done through the soil, foliar, and seed coating but the response to added Zn more pronounced when soil application was done.

Based on the previous literature the zinc deficiency in soils is alarmingly increasing in both global soils and India. The extent of Zn deficiency in soils varies from 30 to 70% in some locations that warrant immediate attention. To alleviate Zn deficiencies in crops, several strategies have been initiated across the world. Already we attempted to develop controlled-release fertilizer of zinc fortified by a manganese core–shell and nano-zeolite based zinc fertilizer that helps in control release fertilizer for rice crop^34,35^. The carbon sphere-based nano-zinc fertilizer was new in agricultural fertilizer industry so considering this point and examined in this study.

## Materials and method

The chemical like glucose, unadulterated zinc metal molecule, zinc sulfate, Polystyrene Sulfonate (PSS) Polyallylamine Hydrochloride (PAH) are bought from Bangalore (Sigma Aldrich, Private Limited), were utilized as crude materials in this examination.

### Synthesis of carbon sphere and fortification of Zinc

Reasonably pure Zn metal (1 μm in diameter) and glucose were utilized as crude materials for the combination of the carbon sphere. In tempered steel autoclave, 17 g of zinc metal pieces and 7.8 g of glucose were included in the wake of blending in a measuring vessel. The materials in the autoclave are fixed firmly and warmed at 460 °C in the electric stove for 8 h then the items are cooled and gathered and the residuals are expelled by treating with hydrochloric acid and distilled water at that point dry the substance for 10 h at 50 °C to get dark colour powder.

To prepare slow-release fertilizer the carbon sphere is fortified with 1.0 M zinc sulfate for 8 h and filtered, by then it is washed with distilled water for 3–5 times and shade dried. To blend of zinc-loaded carbon sphere the solid and liquid extent is 1:10 ration was used. Correspondingly the measure of zinc sorbed was evaluated from the difference between the initial and the equilibrium solution concentrations. Finally, moderate release nano zinc loaded carbon sphere was described by using the diverse instrument for consistency. The physicochemical properties of the carbon sphere and Zn loaded carbon sphere are given in Table 1.

**Table 1:** Physical and chemical properties of carbon sphere.

Parameters	Carbon sphere
**Physical properties**	
Size (nm)	160.6–173.2
Shape	Round
Surface area (m^2^ g^−1^)	9.75
Bulk density (Mg m^−3^)	0.35
Color	Black
**Chemical Properties**	
pH (1:2 ratio)	7.5
EC (dSm^−1^)	0.21

### Experimental soil

The soil gathered from the Tamil Nadu Agricultural University wetland was utilized for this investigation to know the real field conditions. The soil example was collected from the test field in the surface layer (0–15 cm) before the beginning of the analysis and the surface soil tests gathered were spread and air-dried in shade for a time of 5 days and the soil was broken by a wooden hammer, the powdered soil was then filled into plastic pots. Each pot was loaded up with 10 kg of soil. The soil was completely portrayed for different parameters utilizing standard methods. The pH and electrical conductivity were resolved from water concentrates of the soil arranged from a 1:2.5 ratio soil: water suspension utilizing a pH meter and conductivity meter individually. The percent of organic carbon and different zinc fraction was studied by using the technique. The available Zn content was analyzed with 10 g of soil sample was shaken with 20 ml DTPA extractant (13.1 ml triehanolamine, 1.967 g DTPA and 1.47 g calcium chloride combined, made up to 1 L and pH acclimated to 7.3) for 2 h and filtered through Whatman No. 42 filter paper and fed into Atomic Absorption Spectrophotometer (Varian Spectra AA 220). Different soil properties appear in Table 2. The soil was mixed and simulate the pot culture experiments to study the release of zinc from the zinc loaded carbon sphere.

**Table 2:** Initial characteristics of the experimental soils.

Properties	Wetland soil
Clay (%)	34.8
Silt (%)	23.4
Fine sand (%)	29.6
Coarse sand (%)	11.7
Textural class	Clay loam
pH(1:2.5)	7.8
EC (dSm^−1^)	0.18
CEC cmol (p^+^) kg^−1^	18.6
Bulk Density (Mg m^−3^)	1.45
Particle Density (Mg m^−3^)	2.65
Organic carbon (g kg^−1^)	8.1
Available nitrogen (kg ha^−1^)	156.2
Available phosphorus (kg ha^−1^)	16.4
Available potassium (kg ha^−1^)	410.5
DTPA Zn (mg kg^−1^)	0.25
**Zn fractions** (mg kg^−1^)	
WSEX-Zn	0.25
OC-Zn	1.31
MnO_2_-Zn	1.76
Amox-Zn	2.57
Cryox-Zn	2.84
Res-Zn	3.52

### Percolation reactor for nutrient release

**Figure 1:**
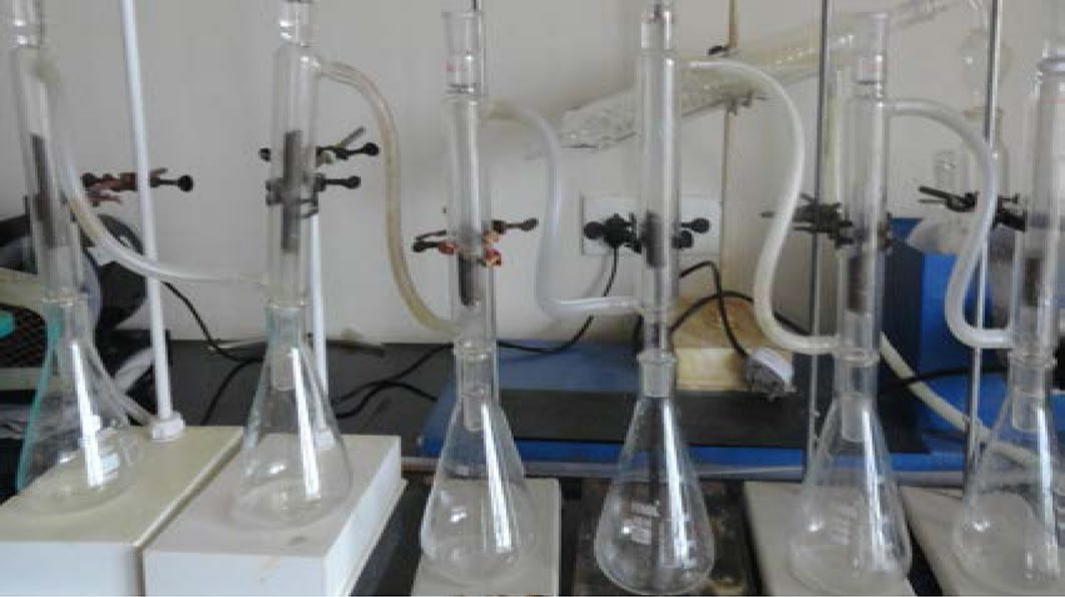
Experimental setup of the percolation reactor for nutrient uptake.

The percolation reactor comprises of (Fig. 1) (interior distance across- 1.5 cm, stature—25 cm) through the highest point of which distilled water is ceaselessly pumped at a stream pace of 72 ml for each day. Inside the permeation reactor, 10 g of the exploratory soil and 1 g of carbon sphere loaded with zinc sulfate overlaid. The Solution was gathered to decide zinc content. The mean temperature during the analysis was 25˚C. A test was likewise performed utilizing pure soil with ZnSO_4_. Parallel reactors were set up to perform the tests in duplicate, and normal values are recounted.

## Results and discussion

### Raman spectroscopy

**Figure 2:**
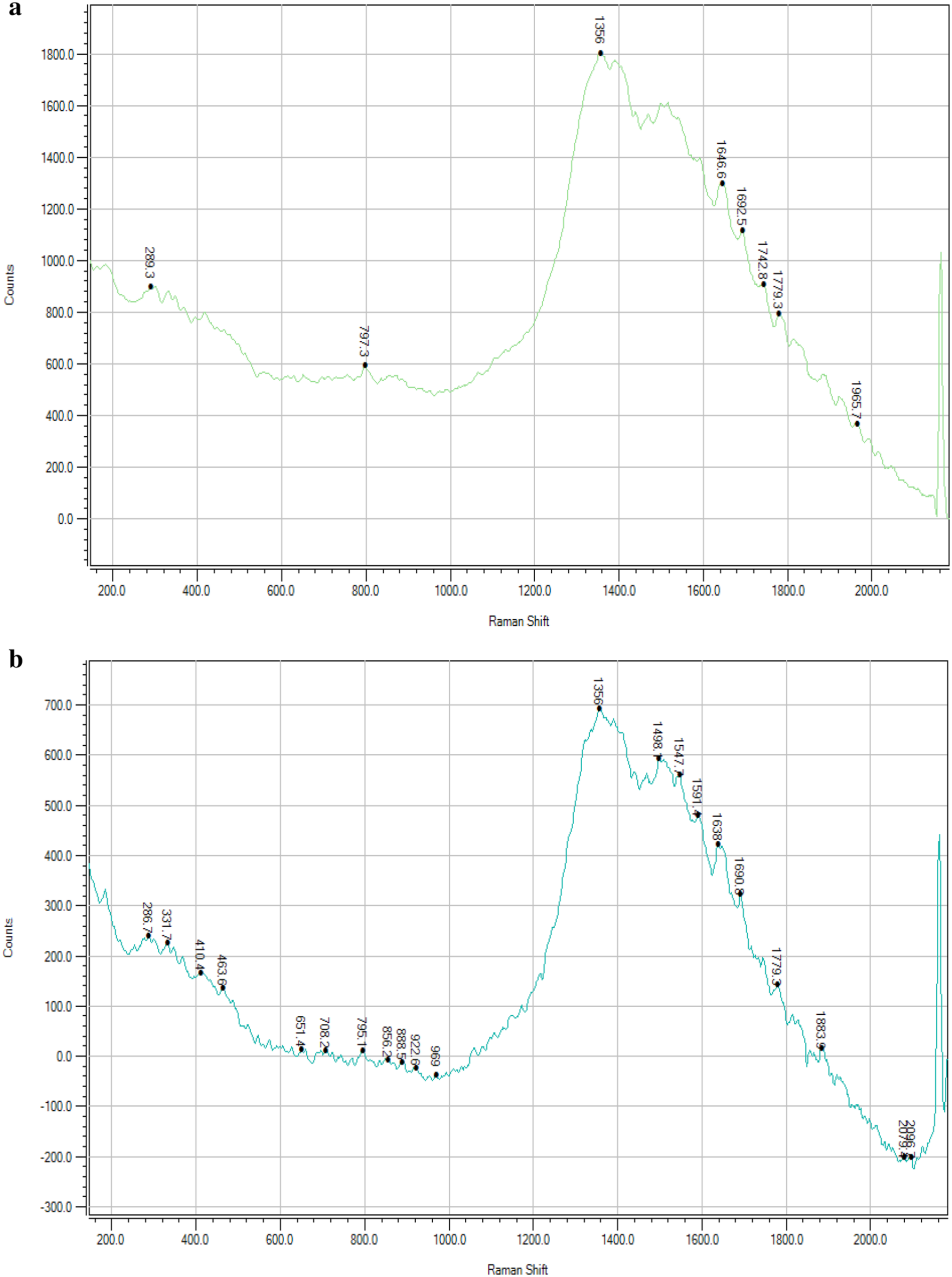
(**a**) Raman spectroscopy of carbon sphere before loading of Zn. (**b**) Raman spectroscopy of carbon sphere after loading of Zn.

Raman spectroscopy technique is based on inelastic scattering of monochromatic light, typically from a laser source. The frequency of photon in monochromatic light changes upon when it interact with sample this process is called as Inelastic scattering. The sample absorbs photons of the laser light and reemits it, frequency of the reemitted photons is shifted up or down in comparison to original monochromatic frequency. The spectroscopic technique normally used to regulate vibrational modes of molecules. Raman spectra for carbon sphere (Fig. 2a) and zinc loaded carbon sphere (Fig. 2b) were recorded peaks at 289.3, 797.3, 1356, 1646.6, 1692.5, 1742.8, 1779.3, 1965.7 cm^−1^ and 286.7, 331.7, 410.4, 463.6, 651.4, 708.2, 795.1, 969, 1356, 1498.1, 1547.4, 1638, 2096 cm^−1^ respectively. The zinc was loaded successfully and it is confirmed with the peak value of 331.7 nm because the carbon sphere having more number of negative charge in case of zinc fertilizer contain Zn^2+^ this divalent zinc cation is easily adsorbed with carbon sphere anion. This result clearly demonstrated that the Zn loaded in nano- carbon sphere can be employed as an effective carrier to fortify zinc sulphate to develop nano-fertilizer formulations. These forms of fertilizers are capable of releasing nutrients gradually and steadily that ultimately enhanced the Zn use efficiency of loaded fertilizer formulations.

### Particle size analyzer

**Figure 3:**
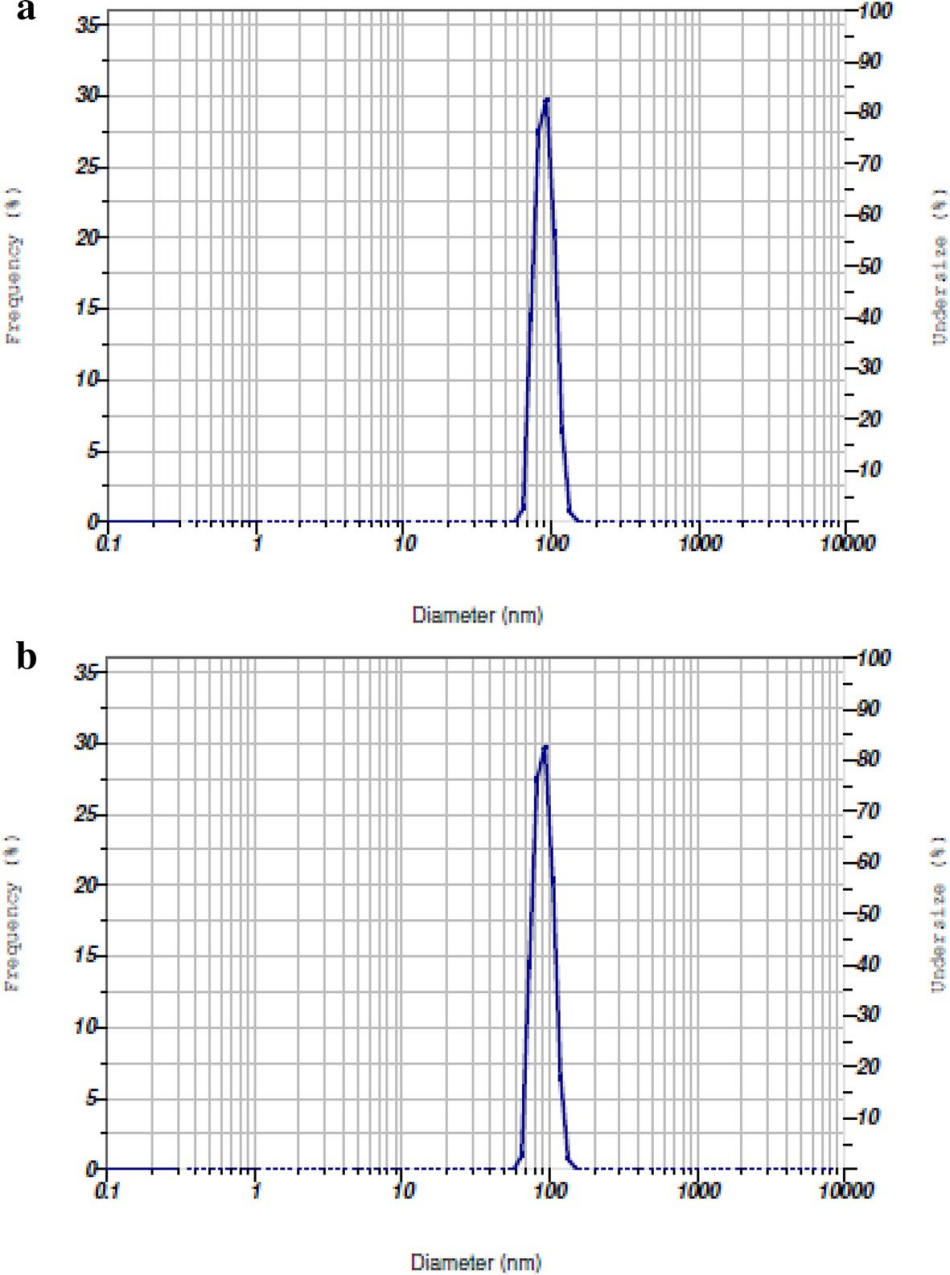
(**a**) Particle size of nano zeolite before loading Zn. (**b**) Particle size of nano zeolite after loading Zn.

The size distribution in the given sample is characterized by particle size analyzer. Several methods are used to measure particle size. The principle of this method is when a beam of laser light is scattered by a group of particles; the angle of light scattering is inversely proportional to particle size (ie. The smaller then particle size, the larger the angle of light scattering). This method is used to measure over a wide size range, very fast, reliable and reproducible technique. The results exposed before (Fig. 3a) and after loading of zinc (Fig. 3b) in carbon sphere values were consistently larger after loading 115 nm than before 100 nm. The result conformed zinc sulphate were loaded in the carbon sphere carrier.

### Scanning electron microscope with EDAX

**Figure 4:**
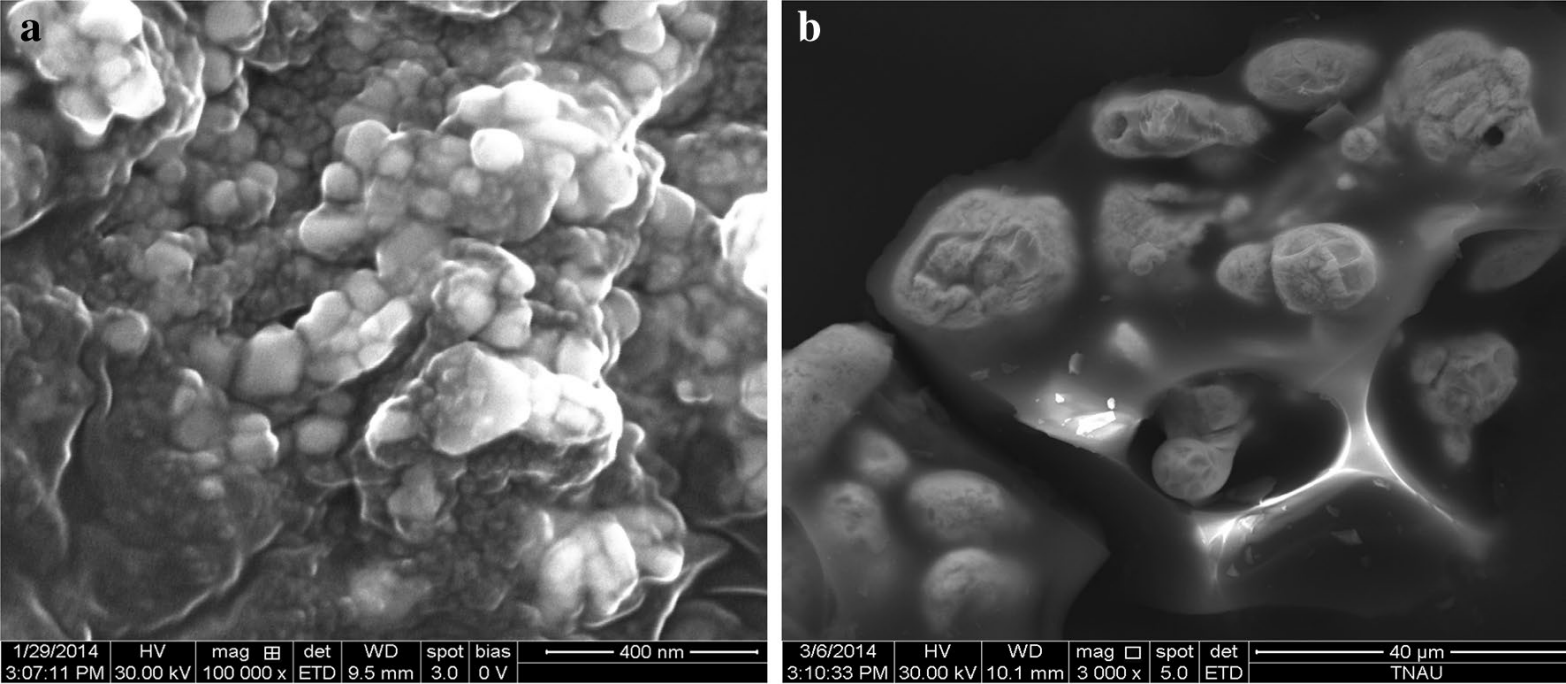
(**a**) SEM image of carbon sphere before loading. (**b)** SEM image of carbon sphere after loading.

**Figure 5:**
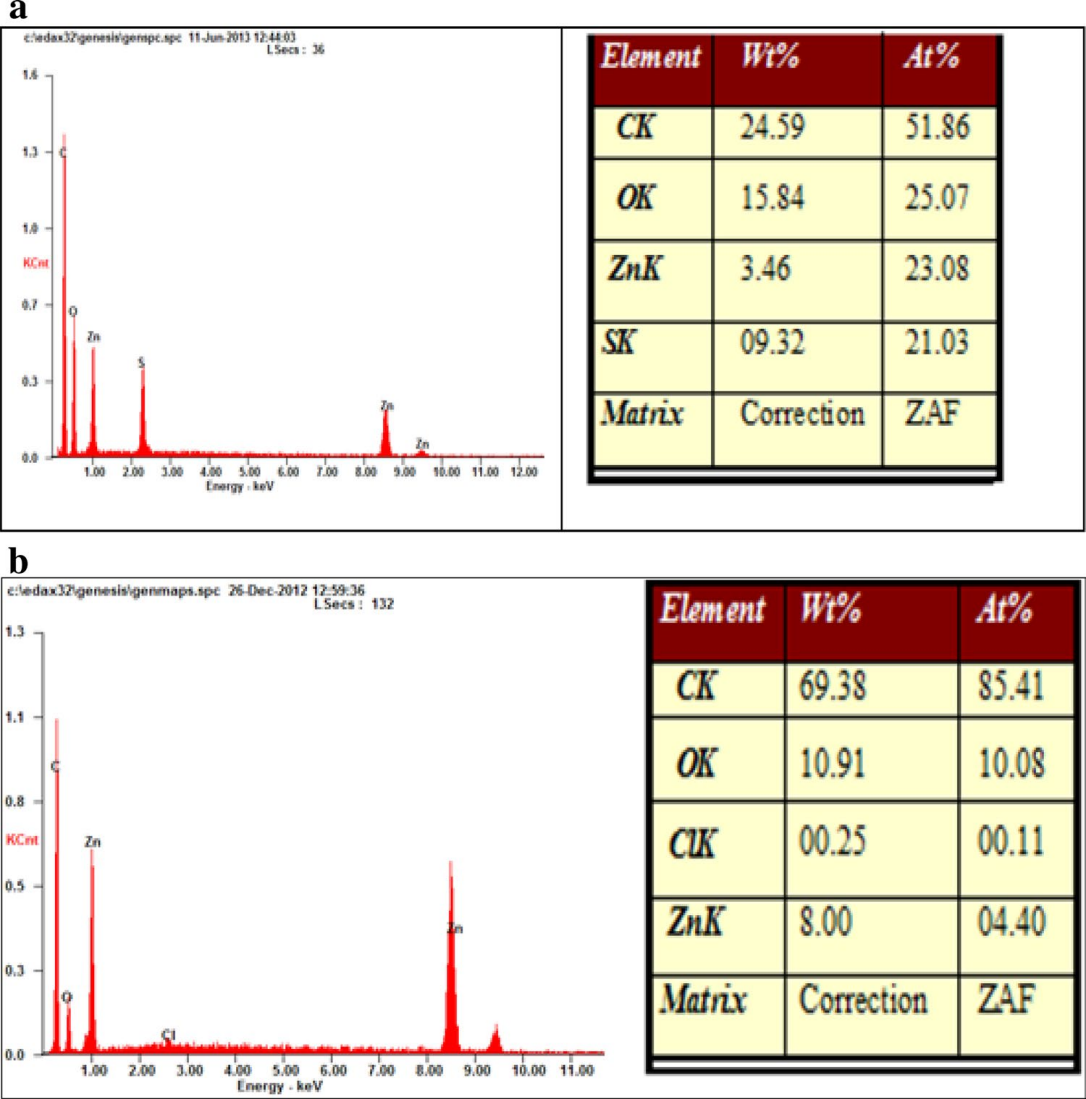
(**a)** EDAX image of carbon sphere before loading Zn. (**b**) EDAX image of carbon sphere after loading Zn.

Scanning Electron Microscope (SEM) is used to acquire data about the exterior structure of the given sample. SEM uses focused beam of electron as an alternative of light to picture the sample. An electron source is used to form a beam of electron, a positive electrical potential accelerate it to a sample. A metal aperture and magnetic lenses confine and focus the electron beam in to a thin monochromatic beam. The electron interact with atom of the sample and produce signals that comprise information about surface topography, composition and other electrical properties. These interactions and consequences are detected and changed into an image. The elemental identification and quantitative compositional data are gathered by using Energy Dispersive X-Ray Analyzer (EDAX). Zinc fortified carbon sphere demonstrated (Fig. 4a,b). The SEM pictures showed that the morphology of the carbon sphere was round like structure before and after loading of zinc. The adsorption of zinc ions on carbon sphere was confirmed by EDAX image and gives composition of carbon sphere. The EDAX pictures given the mineral content of carbon sphere (Fig. 5a) composed of carbon (24.5%), oxygen (15.8%), zinc (3.4%) and sulfur (09.3%) and when zinc was loaded in the carbon sphere (Fig. 5b), the mineral organization had altered to carbon (69.3%), oxygen (10.9%), chlorine (00.2%) and zinc (8.00%). The carbon sphere-based nano-zinc fertilizer contain 8.0% zinc was confirmed by EDAX image^36,37^.

### Zeta potential

**Figure 6:**
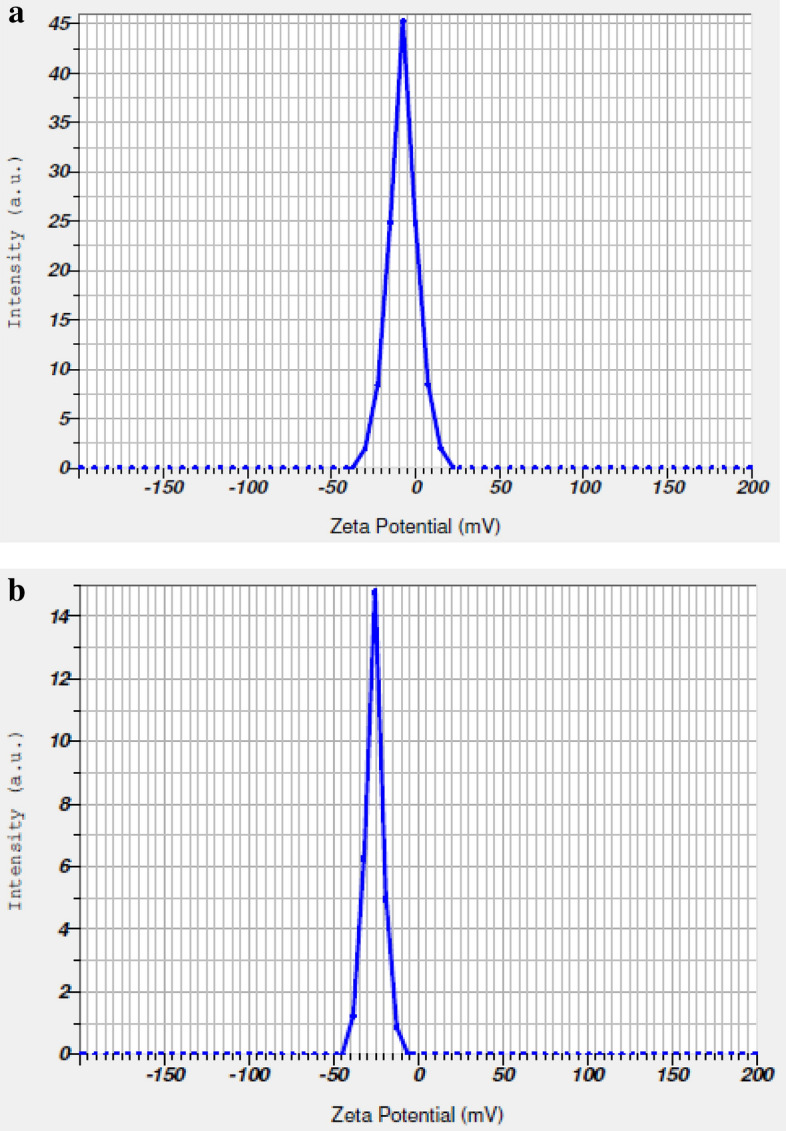
(**a**) Zeta potential of carbon sphere before loading Zn. (**b**) Zeta potential of carbon sphere after loading Zn.

The zeta potential was characterized using a zeta analyzer which displayed that the carbon sphere had the surface negative charge of (−) 45.2 mV (Fig. 6a) and zinc fortified carbon sphere (−) 49.6 mV (Fig. 6b). After Zn loading, the negative charge decreased because of more Zn^2+^ attached to surface of the nano-zeolite. The zeta potential of zeolites depends not only on the pH, but also on ionic strength of the suspension, and the Al content of the framework. According to the DLVO (Derjaguin Landau–Verwey– Overbeek) theory, a potential barrier between surface charged particles in suspension may result in colloidal meta-stability These findings closely substantiate with the reports of^38^.

### Surface area and porosity of carbon sphere

**Figure 7:**
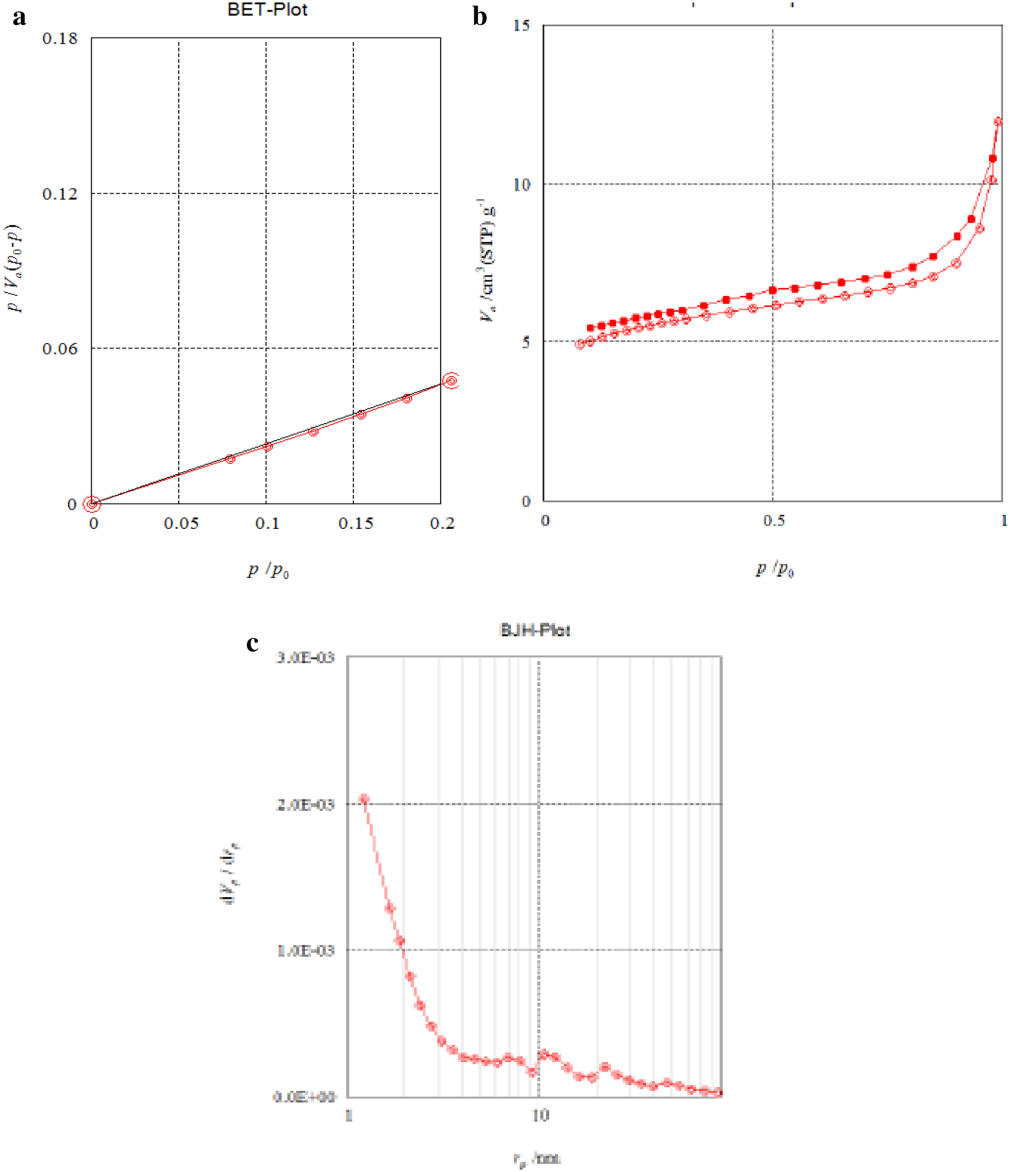
(**a)** BET-plot of Zn loaded carbon sphere. (**b**) Adsorption/desorption isotherm of Zn loaded carbon sphere. (**c**) BJH pore size distribution curve of Zn loaded carbon sphere.

As indicated by the BET (Brunauer, Emmett and Teller) technique investigation surface zone of Zn loaded carbon sphere (9.75 m^2^ g^−1^) individually. The Zn loaded nano-fertilizer test indicated mesoporosity attributes as it is obvious in the adsorption and desorption isotherm, which is appeared in. The somewhat improved uptake of zinc loaded nano fertilizer that had P/P0 values more prominent than 0.5, individually. The isotherm shows a little level of hysteresis, demonstrating the nearness of some mesopores and the chance of fine accumulation (Fig. 7a,b).

The Barett-Joyner-Halenda technique (BJH) model created in 1951 which depends on the Kelvin condition and rectified for multilayer adsorption is most broadly utilized for computations of the pore size dissemination in the mesoporous and part of the macroporous run. The BJH pore size dissemination bends got from the adsorption information of the isotherm demonstrated a conspicuous pinnacle of carbon circle 10 Å, separately showing the age of mesopores (Fig. 7c). The Zn loaded nano-transporters pore explicit surface region of carbon circle is 4.56, m^2^ g^−1^) and pore volume (0.0116, cm^3^ g^−1^) and small scale pore span (1.2 nm), separately^38,39^.

### Transmission electron microscopy

**Figure 8:**
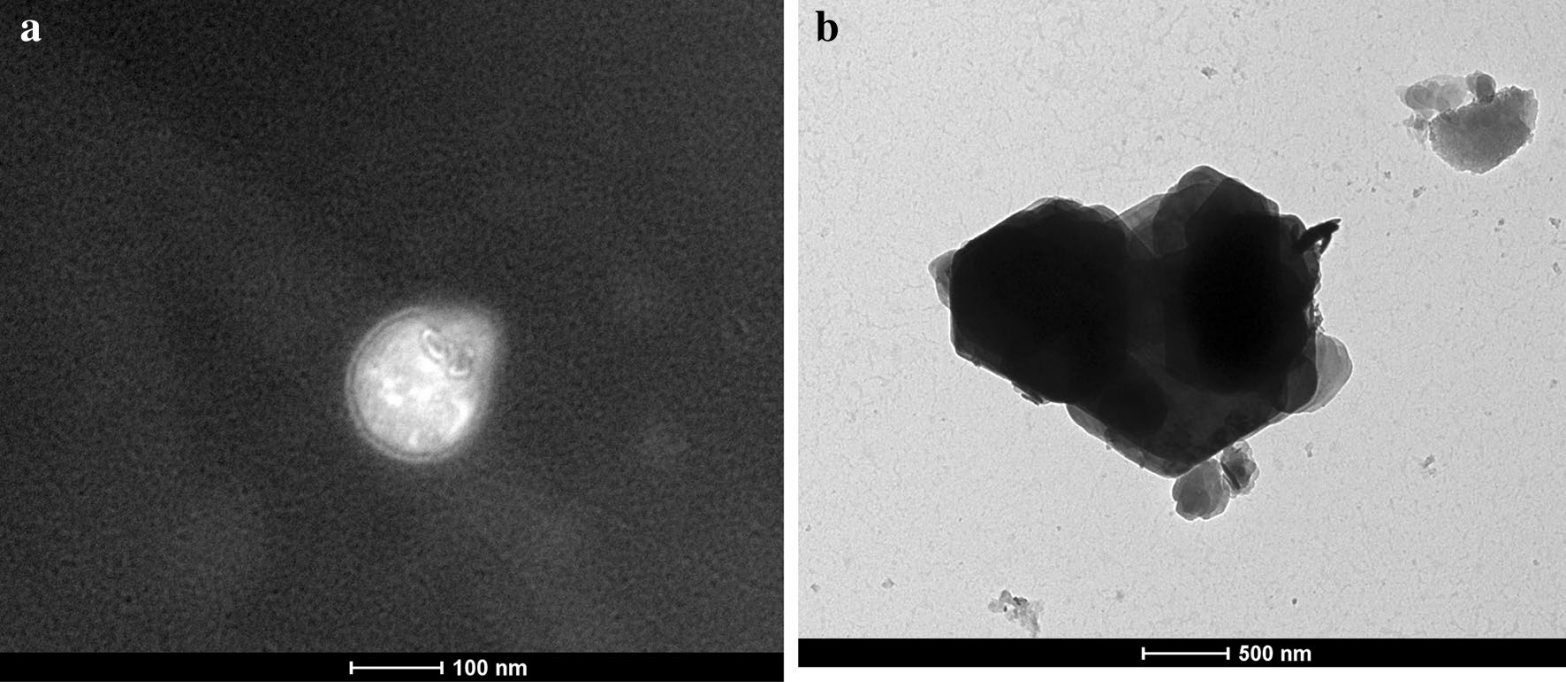
(**a**) TEM image of carbon sphere before loading Zn. (**b**) TEM image of carbon sphere after loading Zn.

The nano carbon sphere was scanned using TEM to determine the internal structure and precious crystal size determination. It showed that the carbon sphere was typically round in shape. The Zn adsorption in the internal surface area of the nano-substrates have been exhibited in the TEM images. The hollow core shell after attaching Zn ions is depicted in Fig. 8b. the TEM image showed that after loading of zinc in carbon sphere the size of the particle get increased (100 nm; 500 nm) (Fig. 8a,b). The Zn is adsorbed in the interior surface region of the carbon sphere. This result was similar to that of^40,41^.

### X-ray diffraction

**Figure 9:**
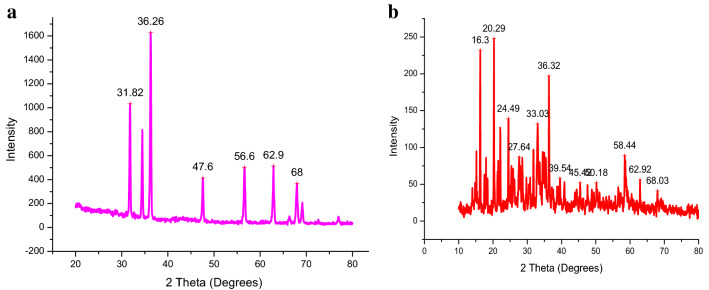
(**a)** XRD image of carbon spher before loading Zn. (**b**) XRD image of carbon spher after loading Zn.

The structural variations of nano-carbon sphere and Zn loaded carbon sphere were determined by using X-ray diffraction (XRD). The XRD of carbon sphere at 2θ = 31.82, 36.26, 47.6, 56.6, 62.9 and 68 degree was observed (Fig. 9a) while zinc loaded carbon sphere (Fig. 9b) had at 2θ = 16.3, 20.29, 24.49, 27.64, 33.03, 36.32, 39.54, 45.42, 50.18, 58.44, 62.92, and 68.03 degrees. The maxima peak of zinc loaded carbon sphere (2θ = 36.32°; d dispersing = 2.47) which matches with the index (JCPDS card No.01–0792)^42^^.^ Overall, our data clearly demonstrated that Zn loading in carbon sphere carriers truly reflected and coincided with the standard catalogue values. Further, d spacing had decreased with Zn loading confirming the successful synthesis of carbon sphere fertililzer formulations.

### FT-IR spectra

**Figure 10:**
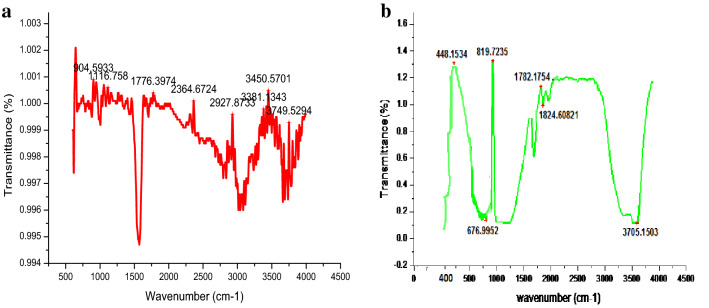
(**a)** FT-IR spectra of carbon sphere before loading Zn. (**b**) FT-IR spectra of carbon sphere after loading Zn.

The analysis of FT-IR spectra showed the presence of functional groups and indicative of proper modification. The FT-IR spectra of carbon sphere having peak value 904.59, 1116.75, 1776.39, 2364.67, 2927.87, 3381.13, 3450.57, 3749.52 cm^−1^ (Fig. 10a). The (Fig. 10b) indicated the IR spectra of carbon sphere loaded with Zn wavenumbers 448.15, 676.99, 1782.17, 1824.60, 3705.15 cm^−1^ in the area of 4500 to 400/cm. The analysis of FT-IR spectra displayed the occurrence of functional groups and revealing of proper alteration^43^_._ The IR pattern of the carbon sphere loaded with zinc exhibited the characteristic peaks at wave numbers 448.15, which is considered to be the zinc peak^44^.

### Zinc sorption of carbon sphere

**Figure 11:**
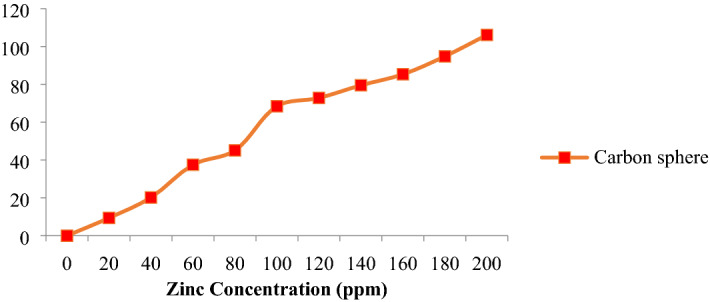
Zinc sorption pattern in carbon sphere.

The carbon sphere was loaded with zinc sulfate of various molar concentrations. The zinc sorption on the carbon sphere demonstrated the measure of zinc sorbed expanded with the increasing equilibrium concentration. The carbon sphere (106.1) sorption at 200 ppm focus of ZnSO_4_ was observed (Fig. 11). The adsorption rate gained with the nano-carbon sphere appeared to be efficient adsorbents for zinc to the rice crop. The adsorption of zinc has been reported by many investigators^45–54^.

### Slow-release of zinc fertilizer

**Figure 12:**
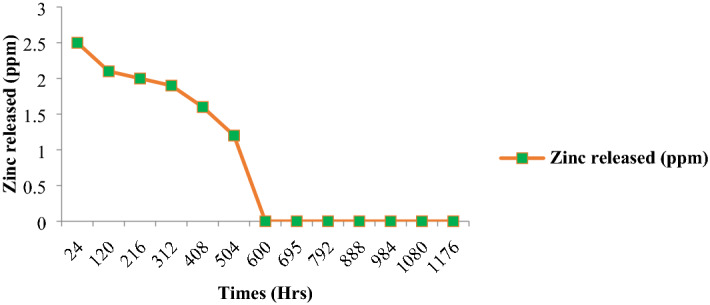
Nutrient release pattern of zinc loaded carbon sphere.

The nano-carrier loaded with ZnSO_4_ was exposed to zinc discharge utilizing the percolation reactors. The percolation reactor with soil and without fertilizer gave just imperceptible measure of Zn. In this way, the entirety of the Zn estimated from the leachate got from percolation reactors having soil plus fertilizer can be attributed to the fertilizer source exclusively. Toward the beginning of the investigation, the highest concentration of 6.1 ppm of zinc was enlisted in the leachate from pure ZnSO_4_ followed via carbon sphere 2.5 ppm. The information uncovered that the entirety of the accessible zinc in pure ZnSO_4_ was depleted after 216 h past which the grouping of zinc arrived at as far as possible. The arrival of zinc from the zinc-loaded carbon sphere proceeded up to 600 h (Fig. 12). The controlled-release fertilizers are used to release nutrient contents of the fertilizer in gradual manner to correspond with the nutrient requirement of a plant. The carbon sphere is excellent plant growth medium for supplying plant roots with additional vital nutrient cations and anions. The process such as dissolution and ion exchange reaction provides the slow release of nutrients in plant root demand driven fashion^55^.

### Zinc loaded carbon sphere and yield response of rice

Under submerged soil situations, zinc fertilization supply through carbon sphere-loaded zinc had improved nutrition, growth, and yield parameters, but the improvement was more distinct and important for shoot zinc content and grain yield (Table 3). The percentage rise in shoot zinc and grain yields were 15 and 31% higher than their respective controls which were fertilized with conventional zinc sulphate. Carbon sphere loaded zinc increased the grain and straw yields by 28 and 25%, respectively. The data clearly shows that carbon sphere-loaded zinc improved the grain yields of both the systems of rice growing^56,57^.

**Table 3:** Zinc loaded carbon sphere application and Yield parameter of rice (*Oryza sativa* L).

Parameters	Submerged		Aerobic	
	Control	Carbon sphere	Control	Carbon sphere
Shoot dry mass (g hill^−1^)	12.57	14.50 (NS)	10.15	10.48 (NS)
Shoot zinc content (mg kg^−1^)	30.42	35.48 **	27.87	30.12 (NS)
Shoot zinc uptake (mg hill^−1^)	3.82	4.32 (NS)	2.82	3.20 (NS)
Grain yield (g pot^−1^)	150.2	210.1 **	127.2	178.1 **
Straw yield (g pot^−1^)	336.8	350.3 **	210.8	283.4 **
Total yield (g pot^−1^)	550.9	590.8 **	446.2	465.3 **

Notes NS-Non Significance.

**Significance.

## Conclusion

The current outcome of the study indicates that the zinc loaded carbon sphere may act as a greater substrate and nutrient transporter for rice crops. It has the properties of a slow-release source of zinc fertilizer and assuring higher yields. The carbon sphere having high surface area leads to increase nutrient holding capacity which minimizes losses of added fertilizer. The results are quite encouraging that nano-forms of Zn such as carbon sphere are capable of releasing slowly and steadily that would have assisted in improved Zn use efficiencies by rice. Thus, it can be used as a nano-fertilizer for crops to improve productivity and reduce the ecological hazard.

## References

[1] Hafeez B, Khanif YM, Saleem M (2013). Role of zinc in plant nutrition. Am. J. Exp. Agric..

[2] Reetu B, Anu K, Salwinder Singh D (2019). Evaluation of Efficacy of ZnO Nanoparticles as Remedial Zinc Nanofertilizer for Rice. J. Soil Sci. Plant Nutr..

[3] Catalina C, Soledad M, Merce L, Berta G, Roser T, Charlotte P (2019). A role for zinc in plant defense against pathogens and herbivores. Front. Plant Sci..

[4] Dimkpa C, Bindraban P (2018). Nanofertilizers: new products for the industry?. J. Agric. Food Chem..

[5] Gokhan H (2020). Zinc (Zn): the last nutrient in the alphabet and shedding light on Zn efficiency for the future of crop production under suboptimal Zn. Plants.

[6] Du W, Yang J, Peng Q, Liang X, Mao H (2019). Comparison study of zinc nanoparticles and zinc sulphate on wheat growth: From toxicity and zinc biofortification. Chemosphere.

[7] Dimkpa CO, Singh U, Bindraban PS, Elmer WH, Gardea-Torresdey JL, White JC (2018). Exposure to weathered and fresh nanoparticle and ionic Zn in soil promotes grain yield and modulates nutrient acquisition in wheat (*Triticum aestivum* L.). J. Agric. Food Chem..

[8] Dimkpa CO, Andrews J, Sanabria J, Bindraban PS, Singh U, Elmer WH, Gardea-Torresdey JL, White JC (2020). Interactive effects of drought, organic fertilizer, and zinc oxide nanoscale and bulk particles on wheat performance and grain nutrient accumulation. Sci. Total Environ..

[9] Rose MT, Pariasca-Tanaka J, Rose TJ, Wissuwa M (2011). Bicarbonate tolerance of Zn-efficient rice genotypes is not related to organic acid exudation, but to reduced solute leakage from roots. Funct. Plant Biol..

[10] Li BY, Huang SM, Wei MB (2010). Dynamics of soil and grain micronutrients as affected by long-term fertilization in an aquic inceptisol. Pedosphere.

[11] Abdul-Kalam, A.P.J. Innovate to Empower Agriculture and Convergence of Technologies. Address and Interaction with the Students, Faculty Members and Staff of the G.B. Pant University of Agriculture and Technology Pantnagar, Uttarakhand on 11 August 2007 (Website <www.gbpuat.ac.in> ) (2007).

[12] Tang W, Zhang Y, Zhong Y, Shen T, Wang X, Xia X, Tu J (2017). Natural biomass-derived carbons for electrochemical energy storage. Mater. Res. Bull..

[13] Zhou T, Cheng X, Pan Y, Li C, Gong L, Zhang H (2018). Mechanical performance and thermal stability of glass fiber reinforced silica aerogel composites based on co-precursor method by freeze drying. Appl. Surf. Sci..

[14] Zhou X, Wan LJ, Guo YG (2013). Binding SnO_2_ nanocrystals in nitrogen-doped graphene sheets as anode materials for lithium-ion batteries. Adv. Mater..

[15] Kim JC (2013). Superior long-term cycling stability of SnO_2_ nanoparticle/multi walled carbon nanotube hetero structured electrodes for Li-ion rechargeable batteries. Nanotechnology.

[16] Liu N, Huo K, McDowell MT, Zhao J, Cui Y (2013). Rice husks as a sustainable source of nanostructured silicon for high performance Li-ion battery anodes. Sci. Rep..

[17] Shen B, Ding J, Yan X, Feng W, Li J, Xue Q (2012). Influence of different buffer gases on synthesis of few-layered graphene by arc discharge method. Appl. Surf. Sci..

[18] Carotenuto G, Longo A, De Nicola S, Camerlingo C, Nicolais L (2013). A simple mechanical technique to obtain carbon nanoscrolls from graphite nanoplatelets. Nanoscale Res. Lett..

[19] Gu LA, Wang JJ, Cheng H, Zhao YZ, Liu LF, Han XJ (2013). One-step preparation of graphene-supported anatase TiO_2_ with exposed 001 facets and mechanism of enhanced photocatalytic properties. ACS Appl. Mater. Interfaces.

[20] Li Z, Wu D, Liang Y, Fu R, Matyjaszewski K (2014). Synthesis of well-defined microporous carbons by molecular-scale templating with polyhedral oligomeric silsesquioxane moieties. J. Am. Chem. Soc..

[21] Jin H, Wu S, Li T, Bai Y, Wang X, Zhang H, Xu H, Kong C, Wang H (2019). Synthesis of porous carbon nano-onions derived from rice husk for high-performance supercapacitors. Appl. Surf. Sci..

[22] Sadhasivam T, Park MJ, Shim JY, Jin JE, Kim SC, Kurkuri MD, Roh SH, Jung HY (2019). High charge acceptance through interface reaction on carbon coated negative electrode for advanced lead-carbon battery system. Electrochim. Acta.

[23] Aziz Y, Shah GA, Rashid MI (2019). ZnO nanoparticles and zeolite influence soil nutrient availability but do not affect herbage nitrogen uptake from biogas slurry. Chemosphere.

[24] Zhang M, Gao B, Chen J, Li Y, Creamer AE, Chen H (2014). Slow-release fertilizer en capsulated by graphene oxide films. Chem. Eng. J..

[25] Liang T, Yin Q, Zhang Y, Wang B, Guo W, Wang J, Xie J (2013). Effects of carbon nanoparticles application on the growth, physiological characteristics and nutrient accumulation in tobacco plants. J. Food Agric. Environ..

[26] Saxena M, Maity S, Sarkar S (2014). Carbon nanoparticles in ‘biochar’ boost wheat (*Triticum aestivum*) plant growth. RSC Adv..

[27] White JC, Gardea-Torresdey JL (2018). Achieving food security through the very small. Nat. Nanotechnol..

[28] Mohanraj, J. Effect of nano-zeolite on nitrogen dynamics and greenhouse gas emission in rice soil eco system M.Tech. Thesis, Tamil Nadu Agricultural University, Coimbatore. **(**2013).

[29] Manikandan A, Subramanian KS (2014). Fabrication and characterization of nano porous zeolite based N fertilizer. Afr. J. Agric. Res..

[30] Sharmila Rahale, C. Nutrient release pattern of nano-fertilizer formulations, Ph.D. Thesis, Tamil Nadu Agricultural University, Coimbatore (2010).

[31] Selva Preetha, P. Nano-fertilizer formulation to achieve balanced nutrition in green gram (V*igna radiata*). M.Sc., Thesis, Tamil Nadu Agricultural University, Coimbatore (2012).

[32] Thirunavukkarasu M, Subramanian KS (2014). Surface modified nano-zeolite used as carrier for slow release of sulphur. J. Appl. Nat. Sci..

[33] Subramanian KS, Sharmila-Rahale C (2012). Ball milled nanosized zeolite loaded with zinc sulphate: a putative slow release Zn fertilizer. Int. J. Indian Hort..

[34] Yuvaraj M, Subramanian KS (2015). Controlled-release fertilizer of zinc encapsulated by a manganese hollow core shell. Soil Sci. Plant Nutr..

[35] Yuvaraj M, Subramanian KS (2018). Development of slow release Zn fertilizer using nano-zeolite as carrier. J. Plant Nutr..

[36] Alizera K, Gholamhosein S (2012). Modification of nano clinoptilolite zeolite with hexa decyl trimethyl ammonium surfactant as an active ingredient of chromate-selective membrane electrode. J. Chem..

[37] Mihaly-Cozmuta L, Mihaly-Cozmuta A, Peter A, Nicula C, Tutu H, Silipas D, Indrea E (2014). Adsorption of heavy metal cations by Na-clinoptilolite: equilibrium and selectivity studies. J. Environ. Manag..

[38] Ferretti G, Galamini G, Medoro V, Coltorti M, Giuseppe D, Faccini B (2020). Impact of sequential treatments with natural and na-exchanged chabazite zeolite-rich tuff on pig-slurry chemical composition. Water.

[39] Danilina N, Krumeich F, Castelanelli SA, Jeroen A (2010). Where are the active sites in Zeolites? Origin of aluminum zoning in ZSM-5. J. Phys. Chem..

[40] Wan W, Su J, Zou XD, Willhammar T (2018). Transmission electron microscopy as an important tool for characterization of zeolite structures. Inorg. Chem. Front..

[41] Esmaeili N (2011). Controlled crystallization of LTA zeolitic nanoparticles from a clear solution using organic template. Iran. J. Chem. Chem. Eng..

[42] Vuonga G-T, Hoanga V-T, Nguyenb D-T, Doa T-O (2010). Synthesis of nanozeolites and nanozeolite-based FCC catalysts, and their catalytic activity in gas oil cracking reaction. Appl. Catal. A Gen..

[43] Guruprasad RA, Yaxue L, Lingkun L, Guiyin F (2017). Synthesis, characterization and applications of microencapsulated phase change materials in thermal energy storage: a review. Energy Build..

[44] Ngoc DT, Thanhand HP, Nguyen KDH (2013). Synthesis, characterization and application of nanozeolite NaX from Vietnamese kaolin. Adv. Nat. Sci. Nanosci. Nanotechnol..

[45] Amir Masoud Arabi E, Rasouli Sousan Arabi S (2011). Synthesis of nano-crystalline Zn pigment by the chemical combustion method: Effect of fuel to oxidizer ratio on structure, microstructure and colour properties. Int. J. Mater. Res..

[46] Abdelaziz E, Najib T, Ihsan S, Kamrul H, Mohamad Al-Farooq K (2019). Characterization of the firing behavior of an illite-kaolinite clay mineral and its potential use as membrane support. Heliyon.

[47] Zhang YG, Gu Y, Wang K, Fang X, Li Z (2012). Fourier transforms infrared spectroscopy approach for measurements of photoluminescence and electroluminescence in mid-infrared. Rev. Sci. Instrum..

[48] Iskander AL, Khald EM, Sheta AS (2011). Zinc and manganese sorption behavior by natural zeolite and bentonite. Ann. Agric. Sci..

[49] Sarkar K, Sen K, Lahiri S (2017). Radiometric analysis of isotherms and thermodynamic parameters for cadmium(II) adsorption from aqueous medium by calcium alginate beads. J. Radioanal. Nucl. Chem..

[50] Mohammad W, Amer M, Fawwaz L, Khalili S, Awwad AM (2010). Adsorption of lead, zinc and cadmium ions on polyphosphate-modified kaolinite clay. J. Environ. Chem. Ecotoxicol..

[51] Bourliva K, Michailidis C, Sikalidis A, Filippidis E, Betsiou S (2015). Adsorption of Cd(II), Cu(II), Ni(II) and Pb(II) onto natural bentonite: study in mono- and multi-metal systems. Environ. Earth Sci..

[52] Sokolova TA, Osipova DN, Ivanova SE, Kiryushin AV (2018). Dynamics of desorption of labile potassium from chernozems. Eurasian Soil Sci..

[53] Zhaohui L, Yingpeng Z, Yan L (2013). Zeolite as slow release fertilizer on spinach yields and quality in a greenhouse test. J. Plant Nutr..

[54] Jumaeri W, Sumarni LW, Ningrum EF, Rahayu M (2020). Using of low grade zeolite based fly ash as slow release agent for *Zea mays* growth. J. Phys. Conf. Ser..

[55] Frantisek M, Macko A (2020). Controlled nitrogen release fertilizer based on zeolite clinoptilolite: Study of preparation process and release properties using molecular dynamics. Curr. Res. Green Sustain. Chem..

[56] Xin T, Xueqin H, Hongwei D, Lujia H, Guangqun H (2018). Evaluation of controlled release urea on the dynamics of nitrate, ammonium, and its nitrogen release in black soils of Northeast China. Int. J. Environ. Res. Public Health.

[57] Muhammad, I., Shahid, U. & Mahmooduzzafar, A. Nano-fertilization to enhance nutrient use efficiency and productivity of crop plants. *Nanomaterials and Plant Potential,* 473–505 (2019).

